# Why do female audiences subscribe to these types of streamers? An empirical study on the motivations of Chinese Huya users

**DOI:** 10.3389/fpsyg.2023.1247451

**Published:** 2023-11-28

**Authors:** Sijun Wang

**Affiliations:** School of Culture & Creative Arts, University of Glasgow, Glasgow, United Kingdom

**Keywords:** game live-streaming, female audience, streamer, uses and gratifications, Honor of Kings

## Abstract

The Chinese live-streaming economy is growing at an accelerated pace among young audiences, but the motivations underlying female users lack academic research. Adopting a mixed approach, this study applies the Uses and Gratification Theory to explain the subscription behavior based on the Chinese live-streaming platform Huya. Through research data collected from online communities (*n* = 202), female audiences’ preferences for streamers has been revealed. Findings show that speech style, humor, and streaming quality are the most prominent attributes, while some results such as excellent mastery of skills, physical appearance, and voice are contrary to previous studies with predominantly male participation. The theoretical and methodological implications of these findings and this approach are discussed. Accordingly, the research gives recommendations to streamers and live-streaming platforms for user growth and maintenance.

## 1 Introduction

Live streaming is a new media-consuming form that began its boom in 2011 (Needleman, 2015), and we are experiencing a time when nearly everyone can produce and consume content online. Among this content, watching others play games is very popular, attracting tens of thousands of audiences per day (Kaytoue et al., 2012). Globally, Twitch is the primary service platform people use, the estimated revenue of which in 2021 has reached 2.6 billion dollars (Business of Apps, 2022). According to CNNIC (2023), as of December 2022, China’s webcasting user scale reached 751 million. Among them, the user scale of the game live broadcast was 266 million, accounting for 24.9% of the total number of Internet users. Industry reports show China’s domestic game market in the first half of this year, with an actual sales revenue scale of 144.263 billion yuan (The Beijing News, 2023). China has become a player to be reckoned with in the live streaming and gaming market. In China, the equivalent service provider is Huya Live, which is also the pioneer in this field. It is a platform containing live streaming content and an online gaming viewing platform. The January to March 2020 report reveals a total of 1.88 billion hours of viewing by users (Huya, 2020), with an average of 85.4 million (Huya Inc., 2021, p. 68) mobile monthly active users of Huya. While the research on game streaming is booming as the watching habits of the public have been established, a few aspects are still underexplored. In particularly, there is a research gap existing in identifying female viewers’ motives for subscribing to specific streamers. Existing studies based on Twitch to detect user motivation present results with predominantly male participants (Sjöblom and Hamari, 2017; Hilvert-Bruce et al., 2018; Shen, 2021). This is in accordance with the social stereotype that male users make up the majority as if they are born to have a preference for and talent in games. However, female audiences ought to be considered since women made up one-third of e-sport streaming viewership in 2019 (Bondy, 2020); the number in China is close to 50%.

To investigate the motives that audiences have for engaging with social media platforms, specific attributes have been included in researches about various media platforms. For example, researches about users’ choices of particular types of content on social networking sites (Hanson and Haridakis, 2008) and on live streaming shopping environments (Sun et al., 2019; Hou et al., 2020) have been conducted. In the study, the relationship between user preference and their engagement is investigated. Essentially, user engagement behavior investigation is about the relationship between platform attributes and user preferences. Noticeably, in the era of mass media, relationship and commitment to media consumption have already been discussed; the founders of Uses and Gratification Theory (U&G) had insight into the relationship between user needs and media. In the new form of live gaming, the interactive attributes are further enhanced. U&G can also be perfectly suited but simultaneously more complex when employed. This study therefore integrates this notion of preference with U&G to form a complete analytical framework. Using this framework, this paper will address the current research gap in the preferences of women in China especially in gaming platforms represented by Huya. Through this approach, the paper focuses on the application of U&G in specific media use scenarios, extending its applicability to the relationship between streamers and audiences in the context of specific content consumption compared with general consumption. Furthermore, due to the differences between the Eastern and Western cultural arenas in which the Twitch platform and Huya are located, the correlation between Para-social Relationships (PSR) and subscription preferences in the context of China will be explored. Therefore, a new and strengthened viewer motivation-streamer attribute analysis model is set up in this research integrating U&G, PSR (which are the core theories), and other existing concepts applicable to user preference to investigate the interaction between users and platforms in the Web 2.0 era.

Based on the discussion above, this study attempted to further understand engaging motivations with regard to gender and focused on the specific types of game live streamers and content on platforms, which was of economic and theoretical importance. Subscription was selected as the primary topic in the present research since it was the first step to analyzing other consumer behavioral patterns and could indicate future pay intention. Chinese female viewers watching Honor of Kings (HOK) on Huya were chosen as the survey subjects. Honor of Kings (HOK) is a real-time multiplayer online battle arena (MOBA) game which was the first mobile game to have more than 100 million daily active users in the world (Zhong, 2021). A mixed research method was adopted. Data from a total of 202 participants were analyzed. Data were collected from quantitative questions and open-ended questions.

The following are proposed research questions:

*RQ1*: What are the most important attributes for female viewers when they decide to subscribe to a streamer?

*RQ1-1*: What reasons and motivations are related to their choice?

*RQ2*: Do men and women have different preferences and motivations?

## 2 Literature review

### 2.1 Uses and gratifications of live game streaming

Uses and gratification (U&G) theory argues that people use media for a certain motive out of cognitive and affective needs and to obtain a particular satisfaction (Katz et al., 1973; Ruggiero, 2000). The satisfaction audiences seek refers to their expectation from the media including information, fun, and other elements. It assumes that the audiences are rational and that they are expecting a certain function or use from the specific media (Wang et al., 2008).

U&G has been used in a wide range of different social media contexts. Ruggiero (2000) contends that the rise of computer-mediated communication has brought uses and gratifications back into sharp focus and can be used to predict the future of mass communication theory. Video game streaming is a completely suitable field for the U&G perspective to be employed. In a global context, Hamari and Sjöblom (2017) led a study to test the foundational motivations of spectatorship by quantifiably measuring gratification from potential aspects including social interaction, learning, and entertainment as shown in former studies (Kaytoue et al., 2012; Hamilton et al., 2014). Hamari showed the impact of five classifications including “Cognitive, Affective, Personal Integrative, Social Integrative, and Tension Release” summarized by West et al. (2010).

The initial studies disclose motivations for the Twitch viewing experience. U&G can be employed to work on questions about viewers and live game streaming as it captures the essence of the relationship between viewers and platforms.

### 2.2 User subscription preference and streamer attributes

With changing communication paradigms and diverse modes of interaction, U&G’s categorization is slightly crude. The path of addressing social networking site usage like Facebook shows requirement of refining. Smock et al. (2011) find that Facebook can be viewed as a toolkit that provides various functions. The usage patterns among different individuals are not uniform. Similarly, the approach of this research to Huya reflects this understanding. The live streaming platform is considered to contribute to content democratization (Pires and Simon, 2015), rather than one-dimensional content delivery. Therefore, when considering Huya, it seems very important to connect specific types of content with use motivations. In real media environments, users will choose to subscribe and follow streamers that can satisfy their expectations most; likewise, Facebook users select functions when they wish to perform different activities and YouTube viewers watch different channels for entertainment or getting knowledge. The question is what attributes make the streamers special and why? However, among all the research on the motivations, most of them start from the point of the streaming platforms. For practical content creators and distributors, there is a lack of understanding of how differences in streamers can influence viewers’ engagement.

More attention should be given to granular aspects of media practice to form a sophisticated understanding. Studies have now found the influence of preferences in more detailed studies, which also provided the basis for the analyses in this study. The social attributes of live gaming platforms are the first to be noticed. When focusing on the community of the platform, friendliness and other attitudes of streamers are mentioned to maintain a healthy relationship between viewers and the platform (Hamilton et al., 2014). Streamers come into the research field as they connect the users and platforms. Building U&G on a socio-based context, Hilvert-Bruce broadened the scope of motivations, adding community-related factors such as social interactions, social support, and a sense of community (Hilvert-Bruce et al., 2018). By capturing psychological and behavioral engagements on four indicators including the amount of time and money spent, their work investigated the effect of social motivations on user engagement. Moreover, in order to analyze the characteristics of streamers, the features and outcomes they represent need to be added before the attributes of streamers are linked to abstract motivations and values. Shen (2021) applied laddering interviews and MEC in her study to construct spectators’ value structures, identifying humor, streamers’ skills, physical appearance, and other attributes to connect U&G values. These also became an important source of theoretical framework for this study. From the early stage of U&G application in live gaming to the inclusion of social relationships in the study, many factors have been mentioned and refined.

**Figure 1:**
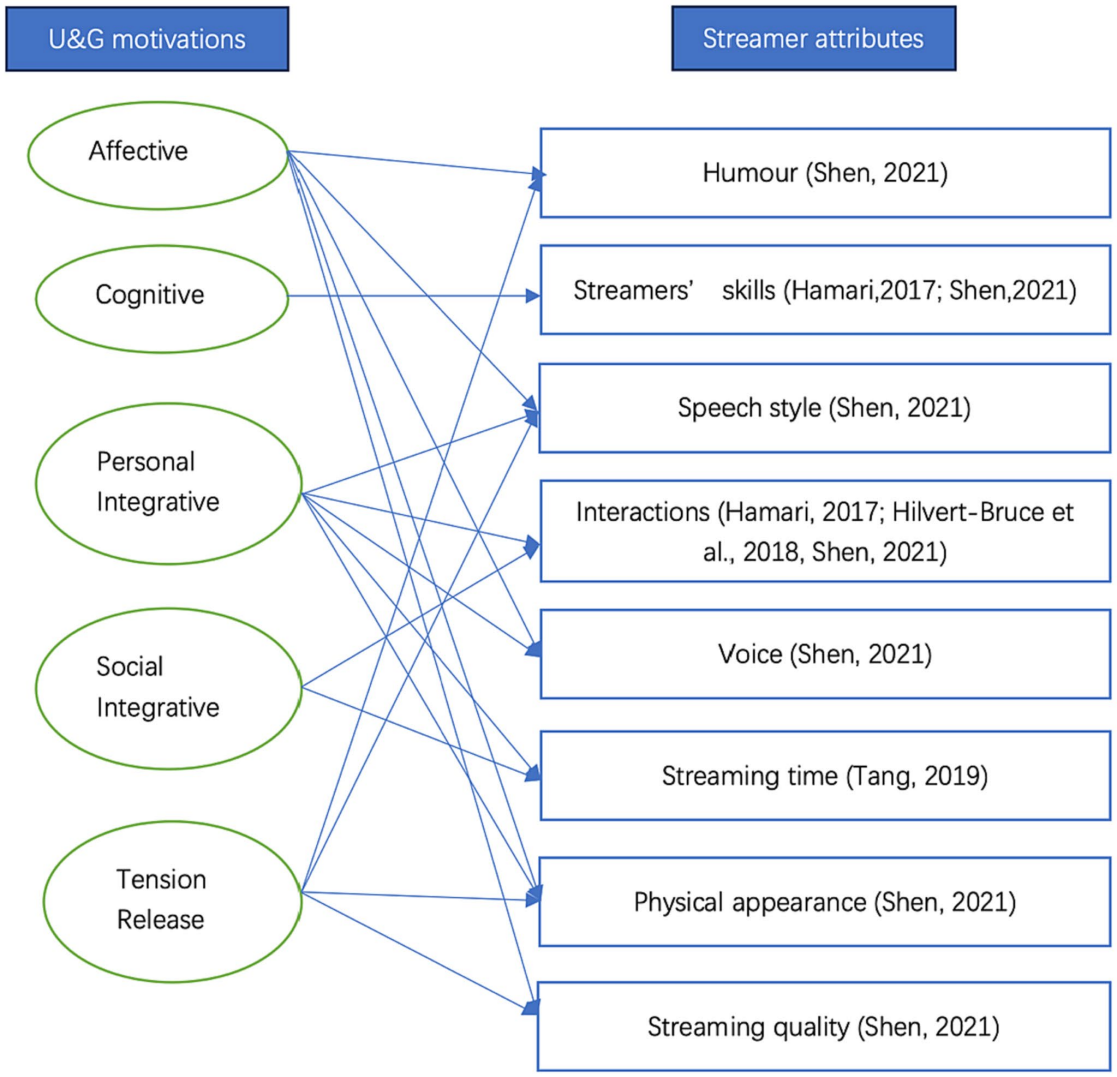
The viewer motivation-streamer attribute analysis model.

Therefore, this research originally proposes a viewer motivation-streamer attribute analysis model (shown in Figure 1) where some common attributes of streamers are modified and matched with the motivations found in previous U&G as a theoretical framework to investigate the relationship between users, streamers, and platforms.

### 2.3 Review of research approach

The research methods used in the study of audience engagement behavior in the field of live broadcasting are summarized in Table 1. From past experience, research methods are mainly divided into quantitative (empirical analysis) and qualitative (empirical materials). Online questionnaires and data crawling can respond to the research topic by summarizing broad trends from macro data. Interviews, on the other hand, can complement single-source data to develop a deeper understanding of the audiences’ personal experiences.

**Table 1:** Review of research methods related to streaming field.

Research topics/themes	Research methods	Advantages	Resource and author
Gift giving/Donation	Online survey	The correlation between behavior and intention can be tested and shown directly	Understanding donation intention in live streaming: a dedication-constraint approach (Chou and Nguyen, 2023)
	Mixed methods (questionnaire + interview)	Data analysis can be verified and expanded, adding depth	Understanding gift-giving in game live streaming on Douyu: an evaluation of PSR/social presence (Zhang, 2022)
	Large-scale dataset capture	It reveals the diversity of paid subscriptions received and made, forming, contributing to systematic understanding.	User donations in online social game streaming: the case of paid subscription in Twitch.tv (Yu and Jia, 2022)
Bullet screens (Danmu)	Big data crawling	The underlying data is very informative	The influence of online Danmu on users’ reward behavior: based on the data of Douyu live broadcast (Huang et al., 2022)
	Big data crawling	Sophisticated models can be developed to analyze the data comprehensively	Exploring the emerging type of comment for online videos: DanMu (He et al., 2017)
	Online questionnaire	The correlation between behavior and intention can be tested and shown directly	What motives users to participate in Danmu on live streaming platforms? The Impact of technical environment and effectance (Wang et al., 2019)
Community	Big data crawling	Describe mass behavioral pattern and predicting model can be created	Modeling and analyzing the video game live-streaming community (Nascimento et al., 2014)
	Ethnographic investigation	Deep firsthand knowledge of stream viewer experience can be developed	Streaming on twitch: fostering participatory communities of play within live mixed media (Hamilton et al., 2014)
	Online questionnaire	Survey data can validate observations from prior qualitative work on Twitch	Who moderates on Twitch and what do they do? Quantifying practices in community moderation on Twitch (Seering and Kairam, 2023)

The discussion in this paper focuses on the data-based analysis of subscription behavior and the specific dimensions discussed subsequently will follow the framework mentioned above. Hamari’s and Shen’s methodological uses also inform the methodology of this study (individual live-streaming participation behavior), such as the use of scales to measure the relationship between motivation and behavior and the use of the MEC chain to decompose the specific performance of participation in live streaming. Also, the open-ended questions allowed participants to give responses other than the alternatives, reducing the influence of the researcher. The advantages of open-ended questions include the possibility of discovering spontaneous responses given by individuals, and biases from previous research can be corrected (Reja et al., 2003), which is in line with the new perspective of women who are the subject of this study. The addition of an open-ended question section provided guidance to refine the design.

## 3 Methodology

Grounded in the systematic review, to focus on concerns about female audiences’ motivations and who they love to watch that has been mentioned in the introduction and literature review, the mixed research method was adopted. A semi-structured questionnaire was chosen to gain a detailed understanding of women’s choices of streamers in response to the research aim of discovering women’s subscription preferences and comparing them with men’s preferences. Questions include quantitative ones, such as scoring the importance of a feature of a streamer. Data and SPSS analysis results could describe the numerical information, finding more specific features of the pattern. On the other hand, predefined quantitative questions have limitations because of the lack of empirical data; open-ended questions were set to welcome emerging ideas and gain rounded information. The two corroborate each other and add to the scientific and objective nature of this article.

### 3.1 Design of the questionnaire

Empirical research has been preferably employed in research on streaming audiences (Sjöblom and Hamari, 2017; Cabeza-Ramírez et al., 2020). Although the Chinese e-sport market is expected to reach 32.11 billion dollars in 2022 according to Chinese consultancy iResearch (Statista, 2022), domestic empirical data is still lacking. Questionnaires have been on the rise since 2017 as an important survey method for game streaming (Harpstead et al., 2019). With a five-point Likert scale, the importance and possibility of attributes affecting their subscription choice will be graded (where 1 = strongly disagree and 5 = strongly agree). Moreover, qualitative data in brief open text format provided an approach to develop knowledge of the detailed conditions and the reason for their grading.

Thus, quantitative and qualitative data were collected. The target population was Chinese female Huya users watching Honor of Kings (HOK) live stream frequently.

#### 3.1.1 Online questionnaire design

Before the questionnaire was made available to a larger audience, a pilot study was conducted (10 female users streaming Honor of Kings) in order to increase the validity and authenticity of the data obtained. Due to the lack of systematic research on whether the streamers’ attributes matter to audiences, especially female audiences, semi-structured questionnaires were designed. In addition to the predefined attribute selected through prior research, Question 16 of part three was provided at the end of the questionnaire for other ideas. In an effort to obtain further rounded information and give participants space to share their feelings, open-ended questions were designed other than grading questions. For example, “Who are your favorite streamers?” can help attract respondents’ interest in case the repetitive grading wears them out.

The final version contains 24 questions in total, including a check question at the beginning. The main part consists of 16 questions, which cover eight attributes identified in the literature review (Qian et al., 2020; Shen, 2021). Inspired by the hierarchical value map (HVM) (Reynolds and Gutman, 1988), most attributes have two or three questions about consequences to thoroughly understand the audiences’ preferred choices in this area. With these derivative questions, tangible or intangible characteristics representing their preferred attributes could be shown.

### 3.2 Data collection

To avoid invalid responses and to find a pool of sufficient participants, the link was distributed through Weibo as well as a few forums and online communities dedicated to games. As a participatory incentive, we provided an opportunity to win a redemption voucher for a hero skin worth 88 yuan or 11 pounds. A lottery was held among all eligible members.

### 3.3 Sampling

The target population is women who watch Honor of Kings (HOK) streaming on the Huya app frequently. For the purpose of enhancing the generalizability of future research, all volunteers are chosen at random. Replies from respondents who misreported their check question, “What is your gender?,” are filtered out as invalid. Additionally, an age limitation was set in the questionnaire. According to the Notice on Further Strictly Regulating and Effectively Preventing Online Video Gaming Addiction in Minors, which was published by The General Administration of Press and Publication of the People’s Republic of China (2021), teenagers are allowed to play video games for 1 h per day on holidays. That means people under 18 have limited experience with gaming and streaming. The responses for Question 1 equal to “under 18 years” are omitted. The final number of valid responses obtained in total is 202. Apart from the five gender-related and four age-related invalid data, the other missing responses reported are non-HOK streamers in Question 3 in watching habit questions.

Participants under 25 comprised 68.93% of all the valid data and only six respondents reported their age to be over 30. This is consistent with the *Huya Annual Big Data Report in 2019* (Huya Inc., 2020), which shows that over 80% of the users were born after 1990. The groups working on or having obtained a bachelor’s degree are the majority (*n* = 127, 61.6%), indicating a relatively high education level. It is noteworthy that the ratio of income under 4000CNY per month (*n* = 97, 47%) is relatively high. This can be inferred that students make up approximately half of the sample. Meanwhile, the majority of the respondents spend over 7 h a week watching streams and have a habit of donating.

## 4 Findings

### 4.1 Basic situation

Table 2 displays the grading results of streamers’ attributes when female audiences decide whether to subscribe to their channels. As mentioned above, the attributes are based on existing research. The top three characteristics of streamers were rated for importance by the 202 respondents: speech style (Average = 4.51), quality of live broadcast (Average = 4.50), and humor (Average = 4.51). Table 3 presents the results of the derivative questions of speech style; of the respondents, 57.77% preferred a peaceful and rational style of speech instead of drama, while 64.08% could not accept the streamer swearing during the broadcast. As Hamilton et al. (2014) found in his research, streamers’ attitudes can influence the atmosphere in the community and their qualities may have an effect on the platform’s openness.

**Table 2:** Grading results on eight attributes of HOK streamers.

Attribute	Number	Min	Max	Average	SD
Excellent skills	202	1	5	4.02	0.92
Humor	202	1	5	4.41	0.72
Interaction	202	1	5	4.28	0.84
Speech style	202	1	5	4.51	0.65
Voice	202	1	5	3.97	0.90
Time	202	1	5	4.13	0.78
Physical appearance	202	1	5	3.81	1.00
Streaming quality	202	3	5	4.50	0.54

**Table 3:** Two derivative questions about speech style.

Question	Option	Number	Rate
What do you prefer: a peaceful mind or a hot temper and dramatic conflict?	Peaceful	119	57.77%
	Dramatic	87	42.23%
Would you accept a streamer swearing in a video game?	Yes	74	35.92%
	No	132	64.08%

The characteristics of the streamers that ranked low in importance were excellent gaming skills (Average = 4.02), voice (Average = 3.97), and physical appearance (Average = 3.81). Table 4 shows the game skills that participants view as suitable. The response rate and popularity of two items, national heroes and all-around anchors, were significantly higher. This result is largely consistent with the ranking of gaming levels.

**Table 4:** Game skill level.

Option	Number	Rate
Professional player	58	20.14%
Single hero ranking top 100	62	21.53%
All-around	82	28.47%
Total score ranking top 10,000	18	6.25%
King level or higher	22	7.64%
Any level	46	15.97%
Total	288	100%

Goodness-of-fit test: χ^2^ = 63.167, *p* = 0.00.

Basically, Table 5 illustrates that audiences usually watch streams at night, which is the same as most streamers’ working time.

**Table 5:** Investigation about watching time.

All day	38	12.34%	18.81%
Morning	31	10.06%	15.35%
Noon and afternoon	44	14.29%	21.78%
Evening	61	19.81%	30.20%
Night	115	37.34%	56.93%
Late night	19	6.17%	9.41%
Total	308	100%	152.48%

Goodness-of-fit test:*χ*^2^ = 117.71, *p* = 0.00.

Responding to pop-up comments, satisfying fans’ requests and lottery are favorite interactions (see Table 6). These kinds of engagement behaviors are commonly observed in streaming platforms and the function of pop-up comments and gift-giving has been identified in prior research (Chen and Lin, 2018).

**Table 6:** Investigation of interactive female audiences.

Responding to pop-up comments	128	27.71%
Satisfying fans’ requests (e.g., try new hero/skin)	152	32.90%
Raffle launching	106	22.94%
Acknowledgement for donations	73	15.80%
Other	3	0.65%
Total	462	100%

Goodness-of-fit test: χ^2^ = 114.73, *p* = 0.00.

### 4.2 Variance and correlation analysis

A variance analysis was conducted to investigate how women with different demographic characteristics had different attribute preferences when subscribing to HOK streamers.

Table 7 illustrates that the difference in preference for the appearance of the streamers when subscribing to an e-sports anchor varied by age (*F* = 10.351, *p* = 0.000). The standard deviation analysis results are shown below. The mean scores of the groups with more significant differences in this factor were “26–30 years old >18–25 years old; 26–30 years old >31 years old and above,” which means that women aged 26–30 are more interested in the physical appearance of the hosts than women aged 18–25 and 31 and above.

**Table 7:** Moderating effect of age on attributes.

	Age (Ave ± SD)			*F*	*p*
	18–25 (*n* = 142)	26–30 (*n* = 54)	>31 (*n* = 6)		
Excellent skills	3.96 ± 0.91	4.22 ± 0.84	3.67 ± 1.51	2.1	0.13
Humor	4.42 ± 0.72	4.44 ± 0.63	3.83 ± 1.47	1.96	0.14
Interaction	4.18 ± 0.85	4.48 ± 0.82	4.33 ± 0.52	2.53	0.08
Speech style	4.53 ± 0.67	4.46 ± 0.64	4.33 ± 0.52	0.4	0.67
Voice	3.89 ± 0.94	4.20 ± 0.81	4.00 ± 0.63	2.42	0.09
Time	4.15 ± 0.71	4.17 ± 0.82	3.50 ± 1.38	2.13	0.12
Physical appearance	3.65 ± 0.99b	4.30 ± 0.84a	3.17 ± 1.17b	10.35	0.00**
Streaming quality	4.50 ± 0.56	4.54 ± 0.50	4.33 ± 0.52	0.4	0.67

*p < 0.05, **p < 0.01.

Table 8 shows that there was no statistically significant difference in the preferences of women with different education levels (*p* > 0.05).

**Table 8:** Moderating effect of education level on attributes.

	Education level (Ave ± SD)				*F*	*p*
	High school or lower (*n* = 14)	College (*n* = 41)	Bachelor (*n* = 127)	Master or higher (*n* = 20)		
Excellent skills	4.36 ± 0.84	4.15 ± 0.94	3.99 ± 0.93	3.70 ± 0.80	1.75	0.16
Humor	4.29 ± 1.20	4.22 ± 0.94	4.46 ± 0.59	4.50 ± 0.61	1.42	0.24
Interaction	4.29 ± 1.20	4.22 ± 0.85	4.28 ± 0.83	4.25 ± 0.64	0.06	0.98
Speech style	4.57 ± 0.94	4.41 ± 0.81	4.54 ± 0.57	4.45 ± 0.60	0.44	0.72
Voice	4.29 ± 1.27	3.93 ± 0.98	3.99 ± 0.85	3.75 ± 0.79	1.01	0.39
Time	4.36 ± 0.84	4.00 ± 0.97	4.19 ± 0.69	3.90 ± 0.72	1.65	0.18
Physical appearance	4.21 ± 0.89	3.83 ± 1.02	3.82 ± 0.99	3.40 ± 1.05	1.91	0.13
Streaming quality	4.64 ± 0.63	4.46 ± 0.50	4.48 ± 0.55	4.65 ± 0.49	0.96	0.41

*p < 0.05, **p < 0.01.

## 5 Discussion

The present study attempts to extend previous research on the motivations for the generic use of live-streaming platforms, with a focus on female audiences’ engagement. By identifying attributes of game streamers preferred by female audiences and clarifying the corresponding consequences they perceive, the viewer motivation-streamer attribute framework is built to provide more insight into the reason from individual and social levels. Adopting U&G as the core theory, the framework aims to refine subscription preference related to specific attributes of the streamers. Through this approach, the user can be more closely linked to the content creator and deliverer; thus, relational use can be more deeply reached. Generally, speech style, streaming quality, and humor were the most important factors. Comparisons with past results suggest gender has an effect on content preference (Sjöblom and Hamari, 2017; Shen, 2021). Particularly, streamers’ game skills and physical appearance play a less important role in their decision. Derivative issues about streamers’ multiple identities under the mixture context are raised in addition to the results directly shown. The results of the study could form the basis for future research into the finer details of live content and streamers, developing more accurate predictions of interactive commitment and economic behavior.

### 5.1 Theoretical implications

Our findings not only reveal the particular types of streamers that win popularity but also provide a deeper insight into the gratifications female audiences seek in terms of streamers’ personal performance and technological facilities when engaging. With regards to the female group, the attributes they voted for and the comparison with the male choices are also clearly presented in the results. The following is a detailed discussion of the theoretical issues.

#### 5.1.1 Speech style, streaming quality, and humor: most favorable attributes of the streamers female users of live game streaming platforms subscribe to

As the three highest-ranked factors are speech style, streaming quality, and humor, respectively, they were viewed as the strongest positive attributes in choosing which streamer to follow. The evidence of their importance and motivation for association can be found in past studies. Firstly, in terms of speech style, Shen (2021) found that watching live games offers a sense of satisfaction and belonging through the opinions expressed by streamers. A number of streamers explain their understanding of game playing and share feelings and viewpoints about game culture (Gandolfi, 2016). From Gandolfi’s view, game streaming is more than a game in that the priority of performance is growing. Streamers’ eloquent outputs play an irreplaceable role in game streaming when it circulates among younger audiences as cultural production since it helps to build a unique attitude and persona as a gamer permeating into their performance. When audiences watch live games, they can gain a different experience from their own games. Secondly, the talk-show-like performance and streamers’ reactions increase users’ enjoyment. There is a correlation between this and humor, which ranks third in the preference factor. Tension release, one of the five classes of gratification, has been proven to have a link with the interesting scenes happening in streaming (Sjöblom and Hamari, 2017; Shen, 2021). Out of a hedonic need, the person in question tends to find streamers who can provide humorous content that they enjoy. Finally, in addition to the content of the broadcast, environmental factors can have an impact on the subscription choices of female viewers. Streaming quality is also in female users’ consideration. High definition, smooth picture, and the right volume can create a comfortable environment for viewers to meet further needs. While the importance of each of these factors has been mentioned before, this study further highlights the prioritization of these attributes over others in the user-streamer relationship through data and ranking.

Beyond the numerical presentation, further explanation is given to refine the motivation for choices about preferences. To begin with, in the discussion on speech, this study extends it by adding the related concepts of socialization and community. When female users are engaging, a distinctive style of language is important for viewers to identify a streamer with his or her own style. By expressing and communicating in a way that is relevant to the subculture with which they wish to engage, viewers and broadcasters convey a sense of authenticity and comfort and bring both sides closer together (Lazzarato, 1996; Duffy, 2017). The audience may develop a strong, positive emotional attachment to someone renowned and become a fan via admiration for their style or creativity (Duffett, 2013). In the live streaming environment, style and creativity are shown in streamers’ eloquence and understanding of the game and beyond. Additionally, the personalized language of the streamer enhances the sense of belonging to the fan community. Game streamers often use a number of game-related terms or buzzwords, which can be effectively spread among gamers who know the game. Sometimes a joke or an action may have a widespread effect and become a Geng,^1^ which is frequently used among the fan base. As Labov (2011) states, language is an important indicator of socially constructed group identity. Geng is difficult to understand by a non-faithful audience and contains the group’s unique speech rules and sign systems. Users without a common discursive context would be excluded from the group while inside the group, bearing similar markers and sharing fan identity may provide a sense of safety (Song, 2022) and a distinctive feeling of belonging (Gandolfi, 2016). The search for belongingness is considered to be the core driver of social interactions and has been revealed to be extremely relevant to computer-mediated communication (Sicilia et al., 2016). In addition, streaming quality is considered more than technical issues. Under this condition, the concept of “Quality of Experience” (QoE) has risen (Bouraqia et al., 2020). QoE not only describes an objective indicator of network performance but also relates to the subjective experience of the user when using it (Zhao et al., 2016). It is an assessment of personal experience in order to satisfy the end-user when interacting with technology and media platforms (Laghari and Connelly, 2012). Therefore, technology can help ensure a suitable objective condition, which is fundamental.

The impact of speech styles on subscriptions provides room for more in-depth discussion, which is an original angle in order to provide a better perspective for analyzing the relationship between the user and the broadcaster. In the subsidiary question on speech style, “Would you accept a streamer swearing in a video game?” innovative conclusions in this area are presented independent of existing research. This discussion will answer the question of what roles female viewers want the broadcaster to take on. Live streaming, as an internet medium, gives the possibility for streamers to take on multiple roles. As Hamari and Sjöblom (2017) argued, gaming, which is seeping into the media, is becoming a more diverse stream form. Are streamers only players or web celebrities? As specifically indicated in the two derivative questions following speech style, women like streamers bearing a peaceful and calm mind during the game rather than having a hot temper and yelling. This may be in line with their aspirations as players: to meet a good-tempered teammate or not to give up so easily. The majority cannot accept impolite words. Answers to why streamers are not expected to swear during live gaming can be summarized into three categories, representing the audience’s expectations of the multiple functions and identities:

1. Swearing reflects an unhealthy attitude toward the game.
2. Swearing can affect the mood of the audience and the outcome of the broadcast.
3. Swearing can damage the image of the streamer and the harmony of the public cyberspace.

The formation of multiple characters may be linked to the close connection between live streaming and gaming, digital media, and related communities (Woodcock and Johnson, 2019). In the first category, streamers were viewed as game players, representing the “general public.” Therefore, streamers belong to the gaming culture, and they are recommended to fulfill their responsibilities as players and remain respectful and passionate about the game. That means female audiences want genuine players engaging with them for a shared love for gaming not for money exchanging hands. Next, streamers are “produsers” of the streaming platform. Evolving from “prosumer” (Toffler, 2022), “produser” is used to describe producing and providing services within people’s interests and expertise (Bird, 2011). “Produsers” may be grass-roots, but their powerful influence can help them go mainstream. The need to satisfy audiences is the priority. As Johnson and Woodcock (2019) argued, live streaming has developed into a new business model based on monetizing content creation. As a new form of entertainment, live streaming can even compete with traditional broadcasting television in terms of popularity. This places certain demands on the quality and professionalism of the content.

Among the fan community, a parasocial relationship represents a relationship between them that is not just content provider and viewer but also star and fan. They, therefore, need to focus on their good image and pay attention to their words and actions since a healthy and positive relationship promotes viewer loyalty and repeat viewing behavior (Lim et al., 2020). Although, originally, streaming platforms such as Huya were for everyone to create and contribute to content democratization (Pires and Simon, 2015), it is actually becoming increasingly capitalized on, as investors recognize the potential to profit from these activities. Streamers need to balance their various roles and responsibilities.

#### 5.1.2 Game skills, physical appearance, and voice: significant divergence in male and female subscription preferences

This section of the discussion is about the three least strong features found in the present study–excellent mastery of skills, physical appearance, and voice, which take a stronger place in male participant-dominated studies.

Firstly, although existing studies have found that information and seeking to improve one’s gaming skills are important motivations for viewers to watch e-sports streaming (Lin et al., 2017; Sjöblom and Hamari, 2017; Shen, 2021), the female users in the present study did not display a significant tendency in this regard. The moderating effect of gender is rarely discussed in this context. Existing empirical studies have a majority of male participants, which corresponds with the gender distribution of Twitch users revealing that 65% of Twitch users are men (Streamscheme, 2023). Commenting on gender differences in live-stream consumption motivation, Todd and Melancon (2018) and Shen (2021) have argued that male participants tend to pay more attention to playing skills. If based on the assumption that improving skills in the MOBA genre is about winning battles, then women’s disinterest in improving skills can be confirmed in Lucas and Sherry’s study (Lucas and Sherry, 2004). Their results suggest that women are less motivated by winning when gaming. Such differences may also be influenced by context and other variables (Cabeza-Ramírez et al., 2020). For instance, geographical differences ought to be considered. Long and Tefertiller (2020) revealed that in China, searching for information is not a significant factor for streaming. It is acknowledged that the level of the players themselves may also have an impact on their attitude to competition and practice. For example, an average-level player may wish to learn more knowledge and movements to improve (Gandolfi, 2016). The limitation is that this data was not collected in the present study. The highest statistical response and popularity rates for “national heroes”(one hero ranking in the top 100 in China) and “all-rounders” were found in the statistics on the level of streamers that women find suitable (see Table 9). These two levels can be thought of as a combination of spectacle and teaching. “All-round” signifies that the streamer is capable of playing all kinds of heroes and modes; thus, viewers can learn a significant amount of information. The “national hero” implies that the streamer has a certain level and skill that satisfies the viewer’s admiration as a fan. It balances challenge and variety without putting up too many obstacles.

**Table 9:** Results of game skill level seen as suitable.

Professional player	58	20.14%
Single hero ranking in the top 100	62	21.53%
All-around	82	28.47%
Total score ranking in the top 10,000	18	6.25%
King level or higher	22	7.64%
Any level	46	15.97%
Total	288	100%

Goodness-of-fit test: *χ*^2^ = 63.167, *p* = 0.00.

Secondly, with regard to voice and physical appearance, it is surprising that the two attributes received the lowest score in the data. Even before live streaming started to boom, a celebrity who is physically appealing in addition to having other qualities (Erdogan, 1999) is believed to be attractive as part of the credibility construct (Ohanian, 1990). In the context of streaming, charming and conventionally attractive streamers can rapidly grab viewers’ attention when showing up on the screen (Chen and Lin, 2018). However, the effect of physical attractiveness alone is limited (McCracken, 1989); for example, it does not have a strong positive effect on purchase intention (Park and Lin, 2020). A combination of other factors such as information can be used to better assess the professionalism of streamers. The numerical information of this research indicates that instinctive requirements cannot generate consistent results of subscription. In fact, viewers may be drawn into a live stream due to the charming cover picture or stay briefly because of a good voice. However, as the viewing time increases, the flow will not be converted into a loyal fan base without the support of high-quality content.

One particular discussion that arose from the appearance question was whether female viewers preferred male or female streamers. Of the participants, 69.8% (*n* = 141) chose male streamers. This may be due to the fact that there are more famous male streamers. Meanwhile, some of the participants’ explanations inspired thoughts about the phenomenon:

*‘Female streamers will use a very heavy filter and retouching, dress slightly revealing, and have a deliberately sweet voice. I’m not quite comfortable with my own cisgender being on screen in such an image. I find it very awkward.’*

*‘It feels like some female streamers are behaving in a contrived manner with male viewers as their target audience.’*

*‘Comparatively speaking, male streamers are more casual and focus more on the game itself. Of course, I would be happy to watch if there were less pretentious girls live!’*

These explanations were related to the resistance of the male gaze (Mulvey, 1989). The term “male gaze” refers to a sexualized way of viewing that empowers men and objectifies and sexualizes women. The development of the internet and the media has accelerated the speed at which images can be distributed as well as the spread of mainstream male-gaze culture (Oliver, 2017). Nakandala et al. (2017) has found that female channels received more comments related to physical attraction and their physical appearance and body while the comment section under male-led channels was more diverse. On the live screen, women may use poses such as “the ironic look of sex kitten, pouty lips, and eyes wide into the camera” (Oliver, 2017, p. 453). The differences are obvious. The gendered bodies may cause “fears of women utilizing their sexuality within an entertainment context” (Taylor, 2019, p. 162). Therefore, the empirical results in this research propose that it is not that female viewers prefer male streamers merely due to their gender or handsome appearance. They want less irrelevance mixed in with game streaming, especially mixed in with content related to sexual attraction, as is predominantly the case with female streamers.

### 5.2 Practical implications

Based on the empirical results and theoretical implications presented in the final section, the author would like to present a discussion and suggestions related to the practical implications of the preference of female viewers for broadcasters. Since women are experiencing growing participation in streaming viewing, this may enable streamers to further drive monetization of the platform while maintaining their own competitiveness and influence in the media market. The following recommendations will be developed from three perspectives: platforms, streamers, and female users.

From the platform’s perspective, as the female user base grows, it is important to have a reasonable balance of preferences for different genders. Johnson and Woodcock (2019) emphasized that “liveness” is a crucial feature of streaming platforms such as Twitch. Diversity of users can help platforms grow healthily and enhance their vitality. As we are in the era of surveillance capitalism, any user actions can be recorded as user-generated data, which will be recorded to make economic income for platforms (Saura et al., 2021). What should be emphasized is that the use of user data is moral and platforms should protect user privacy. As a streamer, understanding the preferences of female viewers can help them create an image that viewers love and create popular content. The following suggestions are given combined with the most important attributes found in the findings. In order to gain audience satisfaction, streamers should practice using appropriate language and giving meaningful opinions. They should be able to illustrate that they are at ease with the terminology, culture, and concepts associated with gaming to ensure that they can present themselves as genuine broadcasters and players (Duffy, 2017). Streamers are “super-individuals” who have been empowered in an unprecedented manner like stars. Streamers should behave themselves and have a positive influence on their fanbases, which can benefit their future branding activities. Having good looks and a good voice is not essential, but it is recommended that the streamers present a natural and clean appearance in front of the camera.

When female audiences repeatedly engage with their favorite streamers or subscribe and develop a periodic viewing habit, they are likely to generate a one-sided, hypothetical intimate relationship with media celebrities, which is defined as a parasocial relationship (PSR) (Rubin and McHugh, 1987). Scheduling meetings, communicating with streamers through comments, and receiving thanks for donations can all contribute to a unique interactive experience. The study’s rank correlation analysis confirms that the amount of time spent watching and donations made are positively correlated with interaction (see Table 10).

**Table 10:** Investigation of the correlations between viewing time and amount of donation with attributes.

	Time spent	Money spent
Interaction	0.19**	0.14*

*p < 0.05; **p < 0.01.

Based on the former discussion, it is recommended that female users should prevent themselves from over-indulging in and irrationally spending money on this kind of online intimacy.

## 6 Conclusion

The present study investigates the preferences of female viewers in terms of their characteristics when choosing to subscribe by developing the viewer motivation-streamer attribute analysis model. Speech style, streaming quality, and humor are found to be the most important attributes for female users and further explanation is given from a social angle. These attributes not only represent streamers’ characteristics but also influence the social interaction between female viewers and streamers as well as viewers and viewers. Meanwhile, preference for game skills, physical appearance, and voice showed relatively large differences between men and women. These differences are closely related to the reality that current live gaming platforms have male users as their main target group.

This research is grounded in the U&G framework to expand the comprehension of female subscription preference and has significant contributions. In particular, this study responds to research calls for a more granular viewer category with regard to gender, nationality, and type of game in future research direction (Hamari and Sjöblom, 2017). Additionally, prior research has discussed the relationship between users and platforms in media use. This work extends previously proposed generic uses by focusing on streamers exploring more hands-on media interaction behaviors and treating interactive live streaming platforms as a “third place” where preferences for streamers are understood in the context of social interactions. Last but not least, the analysis model could serve as a starting point for future studies to find female users’ preferences for other engagement behaviors such as gift-giving.

In addition, different responses of male and female audiences in the framework of U&G caused by gender bias in society and gaming culture are noticed. The difference between male and female users in terms of their usage behavioral characteristics and the way they are treated by the streamers leads to a different parasocial relationship. In essence, this mechanism difference may have roots in the fact that different genders have different psychological needs in consuming live streaming. In this paper, user psychological needs has not been further explored. In the future, adding interdisciplinary theories such as sociology and psychology into the research paradigm to summarize the relationship building between users and anchors as well as loyalty development is expected.

## 7 Limitations

Based on the prior research, the methods adopted in this research have validity and rationality. However, the study is constrained by a number of factors. Firstly, all respondents were from China. The results of this survey may exclusively be of significance in the Chinese market. Data from other countries could be used to refine the findings and test the moderating effect of cultural differences in the future. Secondly, the limited number of participants would weaken the reliability of the results in the entire population. Thirdly, “Zhang Daxian” appeared in most of the responses to the nominated “favorite streamers.” He is the most popular streamer on the Huya platform, followed by 26.42 million fans. However, the high level of repetition and consistent preference of one particular streamer may have decreased the diversity of data. Finally, this study will pre-determine participants’ thoughts during the questionnaire development process and predefine attribute choices in the questionnaire data collection. However, audience psychology and behavior are subjective and more complicated than a uniform set of relevant factors.

## Data availability statement

The original contributions presented in the study are included in the article/supplementary material, further inquiries can be directed to the corresponding author.

## Ethics statement

Ethical review and approval was not required for the study on human participants in accordance with the local legislation and institutional requirements. Written informed consent from the (patients/participants OR patients/participants legal guardian/next of kin) was not required to participate in this study in accordance with the national legislation and the institutional requirements.

## Author contributions

The author confirms being the sole contributor of this work and has approved it for publication.

## Footnotes

^1^Geng is a Chinese word, which means internet joke or punchline.
